# Participants teach nurses how to facilitate forgiveness through lived experiences with media: A qualitative study

**DOI:** 10.1002/nop2.1371

**Published:** 2022-09-25

**Authors:** Ann Gentry Recine, Louis Recine, Ann Aschenbrenner, Shawna Helmuth, Megan Jacobson, Summer Peoples, Nick Walther

**Affiliations:** ^1^ College of Nursing and Health Sciences University of Wisconsin‐Eau Claire Eau Claire Wisconsin USA; ^2^ Holistic Therapy, LLC Eau Claire Wisconsin USA; ^3^ Lou Recine Coaching, LLC Eau Claire Wisconsin USA; ^4^ College of Arts and Sciences University of Wisconsin‐Eau Claire Eau Claire Wisconsin USA

**Keywords:** alternative therapy, art of nursing, arts/literature, health promotion, integrated care, lived experience, phenomenological hermeneutics, psychiatric nursing, psychological and social coping, qualitative approaches

## Abstract

**Aims:**

The first aim was to learn what books and other media the study participants would recommend for inspiring people to let go of anger towards themselves or others and have a kinder intention towards themselves or others. The second aim was to learn which media had these effects on participants, and how. The third aim was to compile lists of cited media that can be available to nurses and other healthcare professionals who want to utilize media therapy for the facilitation of forgiveness.

**Design:**

Phenomenological qualitative.

**Methods:**

Thematic analysis of semi‐structured interviews with 39 participants.

**Results:**

A total of 166 diverse media were identified as useful for helping people forgive, which were compiled into tables and categorized into cross‐culturally useful forgiveness process themes. Findings also verified and expanded a previously compiled forgiveness guidance document that summarized the findings of 11 qualitative studies revealing how people forgive.

## 1 INTRODUCTION

In a media‐filled world, which media do people who are trying to forgive find helpful in their struggle, and why? And are there particular kinds of strategies and resources people use to help them forgive? Forgiveness is not a new concept. Most human beings were probably taught the importance of forgiving others when they were children. With differences in goals, focus and emphasis, forgiving wrongs appear to be international and cross‐cultural (Ho & Worthington, 2018; Obasanjo, 2019). However, despite forgiveness being an internationally practiced belief, professionals need tools for forgiveness facilitation. Implementing forgiveness facilitation interventions when appropriate may help patients and clients experience fewer illness symptoms, a decreased need and use for medication, less insomnia, less fatigue and less depression (Recine et al., 2009).

The cross‐cultural *Nursing Interventions Classification (NIC)* (Butcher et al., 2018) includes two nursing interventions that motivated this study. The first is “Forgiveness Facilitation” (p. 195), which includes a recommended activity for patients to read literature that inspires forgiveness. The second is “Bibliotherapy” described as “therapeutic use of literature to enhance … active problem solving, coping” (p. 84). Coping with being wronged is indeed a problem to be solved. Although scholars have developed processes that help people learn to forgive (Recine, 2015), we are not aware of curated lists of literature or other media that may help facilitate this process. Such lists may be useful to nurses and other healthcare providers who are seeking to help their patients or clients along the path to forgiveness. Through this study, we have compiled what we believe to be diverse lists of media that may be useful to nurses and other professionals who want to implement media therapy as an intervention for forgiveness facilitation in their patients and clients. Many of the media are translated into languages other than English. We have also provided forgiveness‐related categories of literature and other media so that practitioners may be able to develop lists for any language or culture.

## 2 BACKGROUND

**FIGURE 1:**
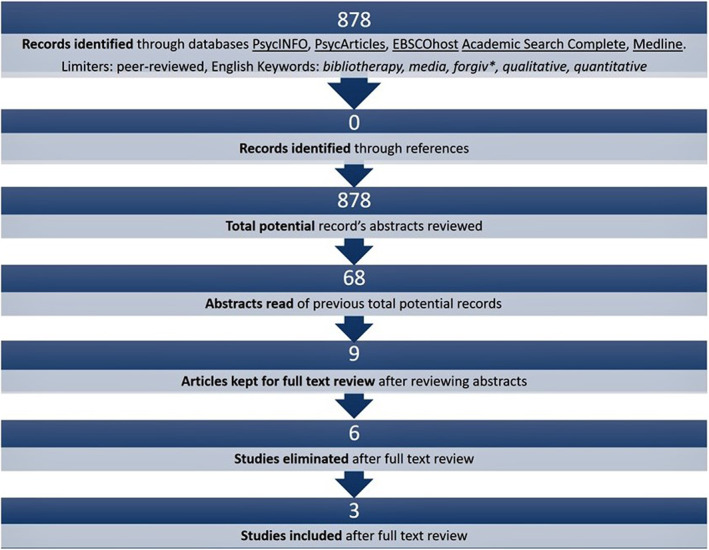
Literature review: What lists of books and other media exist that can help people forgive?

The impetus for this study arose from our literature review to discover whether scholars had compiled lists of books or other media that could help people forgive. Surprisingly, no such lists were found. We searched five databases, reviewing peer‐reviewed English‐language articles (see Figure 1). The review did reveal a radio drama aired in the Eastern Democratic Republic of Congo, which was intended to encourage the forgiveness process after armed conflict and was at least in part effective (Bilali & Vollhardt, 2015). In this, we saw the possible positive effect of other audio media, and films and plays, on the forgiveness process. The discovery of radio drama as useful in the forgiveness process confirmed our decision to include in our study many kinds of media in addition to literature. The review revealed a gap in the scholarly literature that needed to be filled by original qualitative research; a gap which, if filled, might be useful to nurses and other professionals for facilitating forgiveness in patients and clients.

Much previous scholarship has been done on the concept of forgiveness, yielding various definitions. Some examples follow. “Letting go of negative affect…cognitions…and behavior” (Rye & Pargament, 2002, p. 419); “the willful giving up of resentment in the face of another's (or others') considerable injustice” (Baskin & Enright, 2004, p. 80); “fostering the undeserved qualities of compassion, generosity and even love toward him or her” (Enright, 1996, p. 108). Worthington (2009) describes two facets of forgiveness: a choice of the will to stop behaving negatively towards the person who hurt us and growth towards replacing our negative feelings towards the offender with positive ones. Still another definition of forgiveness is that it is a “prosocial motivational change” (McCullough et al., 2003, p. 542). Some of the definitions differ in focus. Some focus on cognitive and volitional changes in the person who has been wronged, while others focus on a process of an emotional change towards the one who hurt us. There is also a scholarly understanding that the process of forgiveness can take time (Gordon & Baucom, 2003; Worthington et al., 2000). Considering these, forgiveness has been defined as “letting go of, or relinquishing, a negative response following an offense and changing to a positive response towards the offender, within a process that occurs over time” (Recine et al., 2007, p. 312). Our study's research questions formed our two primary interview questions and were based on this definition instead of using the word “forgiveness”:

1. “What books, including religious or spiritual writings or other media could you suggest that could inspire someone to let go of anger toward themselves or others and have a more kind intention toward themselves or others?”
2. “From your personal experience, what books or passages from religious or spiritual writings or other media have helped you to let go of negative feelings toward yourself or others and have a more kind intention toward yourself or others?”

Unexpectedly, most of our participants also answered an unasked question during their interviews. At first, we were unsure of what to do with this poignant personal forgiveness process data since we had not asked this question; but then it became the consensus of the research team that answers to the unasked question indeed needed to be included in our analysis and the findings coded since it emerged so naturally as something that the participants as a whole wanted to share. We coded this unasked question as research question three:

- What do you want to share in general, apart from the media, about your personal forgiveness process?

## 3 THE STUDY

### 3.1 Aims

The original aims of this study were trifold. We aimed to learn what books and other media would be recommended for possibly inspiring people to let go of anger towards themselves or others and have a more kind intention towards themselves or others, and from the personal experience of the participants, which books or other media have had these effects on them, and how? Finally, from the answers we received from the study participants, we aimed to compile lists of media that may be useful to nurses, psychologists, healthcare providers, pastors and others who want to utilize media therapy for the facilitation of forgiveness.

It was clear that the aims of the participants as a whole also included sharing their forgiveness process which they had learned even apart from media. So our aim also included analysing this data and discovering how all the data fit into the broader qualitative data on the forgiveness process.

### 3.2 Design

Our research design is based on Heideggerian phenomenology which is an approach that is both true to the interviewees' description of their lived experience and an acknowledgement that the researchers bring their own understanding to interpreting the qualitative data. Heidegger, 1953/2010 says the “truth of being … Ontology is possible only as phenomenology” (p. 33). Heidegger teaches us that to know what is, one starts with experience rather than simply objectifying nature without respect for the subjective human lived experience. Heidegger went further and said that it is not possible to simply bracket oneself and be descriptive in identifying the phenomena, but the human experience is both descriptive and interpretive. We agree that humans cannot hold in abeyance their pre‐knowledge or interpretive mental constructs. The researchers strove to bracket themselves when they endeavoured to describe the depth of the participants' experiences but also brought their previous knowledge of the scholarship concerning the essence of forgiveness to the interpretation of the findings. To elucidate, the interviewers received instruction on the concept and essence of forgiveness according to scholarship. We read and discussed scholarly work on the concept of forgiveness (see Section 2). The Interview Guide document included the request for participants to share media that helped them “let go of anger toward themselves or others and have a kinder intention toward themselves or others” (see Table 2). This wording was to ensure that the participants focused on media that were in keeping with the scholarly understanding of the essence of forgiveness.

### 3.3 Sample/participants

Participants were recruited via convenience and snowball sampling. Inclusion criteria for this study required that participants be 18 years of age or older and were likely to have been exposed to a variety of books or other media. Exclusion criteria were (a) non‐English speaking persons, (b) persons under 18 years of age, (c) pregnant females, (d) prisoners, (e) legally incompetent persons, (f) persons who are not able to give informed consent, (g) institutionalized persons, (h) subjects perceived to be vulnerable to coercion or undue influence and (i) persons with known cognitive disabilities. Criteria (b) through (i) were required by an institutional review board to assure we did not interview people from vulnerable populations.

We advertised the study via social media accounts such as FaceBook, Instagram, Snapchat, posters at businesses and on a university campus and one radio station. We also recruited social contacts who fit the criteria. No remuneration or incentive was offered to the participants. After 39 semi‐structured interviews, no new themes were found so no additional interviews were conducted (see Table 1 for participant demographics).

**TABLE 1:** Demographic characteristics of participants

Name	Age	Gender	Religion	Race
Alyssa	21	F	C	W
Arianna	21	F	C	W
Ashley	21	F	NR	W
Barbara	59	F	C	W
Becca	21	F	C	W
Ben	22	M	C	W
Bill	64	M	C	W
Brandon	20	M	C	W
Carl	64	M	C	A
Carol	73	F	C	W
Cheyenne	21	F	C	W
Devon	22	M	C	AA
Donna	67	F	C	W
Dorothy	67	F	C	W
Fatima	42	F	I	A
George	67	M	BA	W
Hannah	57	F	C	W
Harold	57	M	C	W
Henry	59	M	C	W
Jane	68	F	B	W
Jean	58	F	C	W
John	56	M	NR	W
Judy	59	F	C	W
Kate	31	F	NR	NA
Leslie	21	F	C	W
Louise	27	F	C	W
Margot	23	F	C	W
Maria	39	F	C	NA
Mary	33	F	C	NA
Melody	22	F	C	W
Nicole	25	F	C	W
Nina	49	F	NR	NA
Peggy Lou	68	F	C	W
Richard	63	M	C	W
Samuel	58	M	C	A
Steve	64	M	C	W
Tammy	57	F	C	W
Vicky	67	F	C	W
Walter	71	M	NR	W

*Note*: *N* = 39. Participant names are pseudonyms.

Abbreviations: A, Asian; AA, African American; B, Buddhist; BA, Bahá'í; C, Christian; I, Islam; NA, Native American; NR, No religion; W, White.

### 3.4 Data collection

The data collection was done by a team of eight led by the principal investigator who had previous experience with phenomenological research. The principal investigator taught the team about phenomenological research, prepared an interview guide and trained the team in its use for the semi‐structured interviews. The wording of the initial questions and grand tour questions can be seen in the interview guide in Table 2. Our goal was to do as many interviews as possible in person and if that was not possible due to distance or participant preferences, the interview was done by phone. If the participant preferred, due to their time pressures or communication style preference, we also conducted interviews by email or messaging. A few participants felt they could provide a long list of media or a better explanation of how the media helped them forgive if they responded via email or messaging. Information about the study, including what would be asked of them, was given to participants prior to their interviews. Participants were free to set the time and date of the interview so that they could prepare and reflect on what media helped them forgive. It is clear to us that many of the participants came prepared for their interviews. Questions in the interview guide and the suggested follow‐up questions (see Table 2) were designed to be used by the interviewers to guide the conversation on how specific media helped participants to forgive, based on the essence of forgiveness. All but a few interviews were done in person or over the phone and were audio‐recorded. In‐person interviews occurred in a private venue acceptable to, and convenient for the participant. Data were collected between June, 2019 and November, 2019. The length of interviews varied widely, from 15‐20 minutes to an hour or so.

**TABLE 2:** Semi‐structured interview guide

Initial question	Grand tour question	Probing questions	Additional questions
What books, including religious or spiritual writings or other media could you suggest that could inspire someone to let go of anger towards themselves or others and have a more kind intention towards themselves or others?	From your personal experience, what books or passages from religious or spiritual writings or other media have helped you to let go of negative feelings towards yourself or others and have a more kind intention towards yourself or others?	Can you please tell me more? Can you please explain what you mean by that? How did that happen? What happened next? What do you think is the reason for that?	When that happened [in book, movie, song, etc.] how did you feel? How did that part of the [book, movie, song, etc.] make a difference in your life? How did that help you feel more patient or gentle towards yourself/the person who hurt you? How did [the book, movie, song, etc.,] change how you understand forgiveness? How did that help you let go of negative feelings towards someone who hurt you? What was it was about that character [or part of the book, movie, song, etc.] that gave you what you needed to let of negative feelings towards yourself/the person who hurt you?

### 3.5 Ethical considerations

The study was approved by a university Institutional Review Board. Study participants were provided with a document on university letterhead which included the purpose of the study, a statement that participation is voluntary, a statement that participation shows their consent and how their identity and confidentiality of their data will be protected. The document also provided contact information for the study's Principal Investigator and the Chair of the Institutional Review Board.

### 3.6 Data analysis

The transcripts were initially coded by the team member who did the interview, assigning the numeral 1 for a specific example of media mentioned by the participant, the numeral 2 for where the participant answered our second research question on how that example of media helped them to forgive and the numeral 3 for where the participant described their personal forgiveness process apart from media. The team analysis began with one researcher reading aloud each section of the interview transcript. After the team confirmed the aforementioned numbering, they assigned codes descriptive of the forgiveness process to all sections of data tied to a numeral 2 and a numeral 3. The codes were entered into Microsoft Word Comments in each transcript. In the coding, our goal was to “enter the world of the person and interpret the meaning they assign to the experience” (McConnell‐Henry et al., 2009, para. 14). A few examples of codes are “not taking things personally,” “letting go” and “deliberate process (making a choice).” We sought the experience of continually being “open to new understandings as well to one's own interpretation” (van Wijngaarden et al., 2017, p. 1741). To help facilitate our analysis, we used a Microsoft Word add‐in (Fredborg, 2020) which extracted comments and associated text into a table in a new document.

After an extensive review of all the codes and discussion of various schemas to organize them, we categorized the coded data from how participants answered research question two (about how media helped them forgive), and research question three, under the following forgiveness process themes:

- Experiencing the negative reality of the offence.
- Finding the power to forgive through an intrapersonal source or strategy.
- Finding the power to forgive through interpersonal source or strategy.
- Finding the power to forgive through a transpersonal source or strategy.

Some participants talked about media that helped them to uncover and experience the reality of the hurt. Being honest and clear about the hurt experienced from being wrong is an important part of the forgiveness process (Enright, 2015; Worthington, 2009). Themes relating to how participants found the power to forgive reflected the thematic schema used in Recine et al. (2020). What we mean by “intrapersonal” sources of power are “sources of power within each person that can give a person the power to respond to an offense with forgiveness” (Recine et al., 2020, p. 243). What we mean by “transpersonal” sources of power are “sources of power to forgive, based in something beyond oneself” (Recine et al., 2020, p. 243).

Then in our analysis, through a process of consensus we categorized the media that participants shared with us as follows:

- Media for learning forgiveness through example.
- Media for learning forgiveness through educating oneself about the forgiveness process.
- Media for becoming more forgiving towards yourself.
- Media for becoming inspired and empowered to forgive others.

See Section 5 for more detail. We further categorized the media in our lists in a way that we hope will be useful to practitioners who want to develop their own lists of media for people from a particular culture (see category codes in Appendices 1 and 3–10). These forgiveness‐related categories were determined after the team read aloud together the lyrics and words of each shorter poem, song or quote. In addition, we read synopses together for the longer, adult works to categorize them. The youth books were provided and categorized by age by a study participant who is a children's literature expert, children's book author, children's librarian and a university professor of children's literature (see Appendix 2).

Finally, the team analysed the data from the 39 participants in this present study to see how it fit into the broader nurse‐authored qualitative data on the forgiveness process. The team reviewed the coded data from this present study to see how it compared and contrasted with a systematic review of nurse‐authored qualitative forgiveness research (Recine et al., 2020).

### 3.7 Rigour

The research was led by the first author (a doctoral‐prepared nurse researcher) and the research team included two other doctoral‐prepared nurse researchers, five university undergraduate nursing students and a community‐based life coach. The first author, who educated the team on various qualitative research theoretical approaches and on data collection and analysis, was experienced in phenomenological research.

In‐person interviews ran as long as it took for the participant to respond to the interviewer's questions and to share, if they wanted to, their thoughts about and experiences of the forgiveness process independent of media. Interviews were subsequently transcribed by the interviewer into Microsoft Word documents. Next, the interviewer re‐listened to the interview to verify the accuracy of the transcription. If clarification was needed, the participant was contacted. For our coding and categorization work, we typically met weekly for two hours. Coding and data analysis occurred from September, 2019 through December, 2020. Each code and category descriptive of the forgiveness process was discussed by no fewer than three research team members until we came to unanimity. These discussions helped us to “reduce blind spots” (van Wijngaarden et al., 2017, p. 1741), resulting in deeper, more nuanced coding. We strove to bridle, but not be blinded to our initial impressions and pre‐understandings so that our process of understanding and coding the data did “not happen too hastily or preemptively” (van Wijngaarden et al., 2017, p. 1740).

We strove to provide trustworthy descriptions of our findings, using direct quotations from the participants to help the reader understand the data. The COREQ checklist (Tong et al., 2007) guided the reporting of our findings.

## 4 FINDINGS

The study participants included 26 females and 13 males, with an average age of 46.3, mostly living in the north‐central part of the United States. Thirty‐one identified as Christians, one as Muslim, one as Buddhist, one as Bahá'í and five as having no religion. Thirty‐one participants were White, one African‐American, four Native American and three Asian (see Table 1).

### 4.1 Findings for research question one

In response to the first research question, the participants recommended media they believed to be useful in helping people forgive. For adult‐age books, they identified 34 (including two graphic novels) and for the youth books: 15 picture books, 9 middle‐school books and 11 young adult books. The participants also identified 22 musical works, 5 plays, 14 films and 3 videos/podcasts. In addition, they shared 23 Bible passages, 13 passages from the Qur'an or other Islamic sources, 11 sayings and 6 items in the category of poetry, prayers and precepts (see Appendices 1–10).

### 4.2 Findings for research question two

In answer to research question number two, participants shared their personal experiences with some of the media they said helped them to let go of anger and arrive at a more kind intention towards themselves and others. Below are a few examples of quotes from the study participants describing how the media helped them do this. First below, are quotes that reveal the four forgiveness process themes reflected in the media, followed by quotes that we hope will provide useful media categories. Pseudonyms are used (see Table 1 for demographic descriptions of the participants).

#### 4.2.1 Participant quotes that reveal forgiveness process themes

##### Media about experiencing the negative reality of the offence

Speaking of the song, “Another in The Fire,” by Hillsong United, Nicole said:

That song helps me really forgive the world when I'm like … wallowing in sadness. Because it's okay to feel that way. Like sometimes you just gotta sit there and feel it. Feel unloved, feel grief, feel wronged and then you can start healing after you acknowledge it.

Louise, who gained forgiveness insights from the book, The Subtle Art of Not Giving a F*ck, realized from the book that judging the other person, and deciding how others should, and should not behave, is the response to the offence that causes us to be troubled: “I think a lot of times (not always) the reason we feel angry or upset with someone is because we feel that person acted in a way they shouldn't have, and wronged us in some way.”

##### Media about finding power to forgive through intrapersonal sources

Carol said Ephesians 4, verses 31 and 32 from the New Testament, which says to let go of bitterness, fury, anger and malice within ourselves and to give kindness, compassion and forgiveness to one another, enabled her to more fully grasp the intrapersonal power of choice. She said: “Forgiveness is a choice; anger is a choice.” Ben felt that Brené Brown's book, *The Gifts of Imperfection* helped him with the inner work of self‐forgiveness and added that “it's definitely been helpful in alleviating the stress and anxiety.” He also said that the book *“*taught me how to forgive myself first because you know … you just have to. … your health needs to be there before you can be taking care of others.”

##### Media about finding power to forgive through interpersonal sources

Peggy Lou shared how the Bible passage, Luke 12:48 (about how if you have been given much, much will be required of you) inspired her to draw on interpersonal sources of power. Reflecting on the love she has received from others, she said: “Because I received a lot of love growing up, I am especially responsible to love others. Also because I have been forgiven much, I ought to forgive others.” In speaking of the interpersonal empathy modelled in the play, *Rabbit Hole*, about a woman who tries to befriend a young man who accidentally killed her son, Jane said:

It just emphasizes what a difficult task it is and how much work it takes and how much you really have to make the effort. To forgive you have to really try to see the experience of the other person, to try to guess where they're coming from. You usually don't know exactly what made them behave the way they did. This was an accident but a horrendous one. But generally people's behavior has a lot of meaning and you have to try to dig to understand where a person's coming from.

Leslie talked about the healing power of receiving love from other people in the book, *The Perks of Being a Wallflower*.

She said:

I think this book can really help people let go of anger towards themselves and love themselves … it's okay to be yourself and to be a little broken, because your loved ones will always be there to put you back together, whether they are near or far.

##### Media about finding power to forgive through transpersonal sources

Influenced by the Qur'an, Fatima found the power to forgive through her relationship with God, and shared Al Qur'an # 42:40: “If a person forgives and makes reconciliation he shall be rewarded by God.” Melody pointed out the power of music in her forgiveness process. She said, “Christian worship music is always a great place for me to center my emotions on God rather than my worldly circumstances.” Cheyenne experienced the power to forgive after being raped, from the lyrics of the song, “Praying,” by Kesha: “I hope your soul is changin' … I hope you find your peace.”

#### 4.2.2 Participant quotes that reveal media categories

##### Media for learning forgiveness through example

John saw forgiveness modelling in the world of superheroes in comic books. He said that the heroes “always” choose forgiveness of the villains. “They kind of give them a chance every time for forgiveness.” Maria found an inspiring forgiveness model in a character in the film, *Hacksaw Ridge*. “I need to be better, I need to be Doss,” referring to a Second World War combat medic character in the film. This character was a forgiveness exemplar for her and her whole family. “He was the total image of forgiveness; when they beat him, harassed him and tried to get him to leave and … he was still willing to save 'em after they were like awful to him.” Samuel found exemplars in *Amish Grace*, a film about the forgiving response of Amish families to the mass murder of their school‐age children. He said:

It really shows how it's not easy to forgive. Of course, we have all the theories. We have the Bible passages, and we know how we should behave, but it's not easy. And it's a process. But it shows how it could be done, it should be done.

##### Media for learning forgiveness through educating oneself about the forgiveness process

Brandon discussed how the self‐help book, *Nonviolent Communication: A Language of Life* helped him forgive his father:

It made me really think of what my dad has gone through in his life to make him how he is today. …This has helped with my relationship with my dad. It helped me forgive my father for a lot of things that he did.

Becca gained insights from the book, *I Wish I Could Be There* that helped her forgive her mother. The book educated her about the experiences of a parent with agoraphobia, which helped her empathize with her mother and forgive her for not being able to “be present” for important life events due to agoraphobia: “The entire book really kind of clued into what agoraphobia really is and helped me to forgive in that way.” It also gave her a sense of common humanity: “It makes me feel less alienated.”

##### Media for becoming more forgiving towards yourself

Steve talked about a song about self‐compassion that spoke to him about letting go of self‐blame for circumstances that are out of your control:

The song, “Yours is No Disgrace” talks about people who have been thrown into … bad situations in life … it could be anything … bad family situation … rape … abuse … that when you are thrown into a bad situation it's, it's not about you, it's about something else.

Ashley was inspired towards self‐forgiveness from a song, “Kun Fayakun” (translated “Be and it is”), influenced by a phrase from the Qur'an.

She said:

I'm really afraid of messing up sometimes and I've learned that a lot of other people share that as well. So, when I do mess up and I do make mistakes sometimes, I'm very hard on myself. And a lot of other people are too, but this particular phrase helped me through my journey of self‐forgiveness and self‐acceptance as well.

##### Media for becoming inspired and empowered to forgive others

George mentioned the coming‐of‐age book, *Red Sky At Morning* in which a boy became empowered to cope with his father's death and his mother's racism and drinking. He said:

I think that book would inspire people to realize again that life is very short and you need to just be nice to people and not worry about being angry with somebody or something when it's not going to matter in a few years anyway or even a few weeks from now.

Ben mentioned Brené Brown's Ted Talk, *The Power of Vulnerability*, as a source of forgiveness empowerment for him:

So, then I find myself running into a negative feedback loop of disconnection with what I think is making me feel more connected but is really harming me. … I think she armed me with the mental capacity to handle that.

Devon was inspired to forgive by Matthew 5:1–12, known as the Beatitudes, that teaches the merciful are blessed. He said, “if you believe in the Bible this passage gives you peace in whatever situation you are in.” Fatima was empowered and influenced by a number of passages from the Qur'an and Hadiths about forgiveness which she shared with us, such as: “Let them pardon and overlook. Would you not love for God to forgive you? God is Forgiving and Merciful” (Al Qur'an # 24:22) and “Be merciful to others and you will receive mercy. Forgive others and God will forgive you” (Musnad Ahmad 7001).

### 4.3 Findings for the unasked (unofficial) research question three

Many participants wanted to share their experiences and insights about the forgiveness process even though they did not relate them to media. To honour their desire to share their deeply personal paths to forgiveness we decided to include this “unofficial” question: “What do you want to share in general, apart from media, about your personal forgiveness process?” Similarly to how we categorized our findings for question two we categorized the processes they shared as experiencing the negative reality of the hurt and by interpersonal, intrapersonal and transpersonal sources of power to forgive (see below). Pseudonyms are used (see Table 1 for demographic descriptions of the participants).

#### 4.3.1 Experiencing the negative reality of the offence

Judy gave an example of her awareness of the deep hurt from an offence. She had created a spreadsheet of resources to help her cope with recurrent family emotional abuse. She said, “even with all of the ‘tools’, knowledge and understanding I have acquired and actively use, I was caught off‐guard. The new and deep wounds were shocking and excruciating, and the grief is still fresh.” Hannah described experiencing the reality of the offence. She told the story of her husband's affair and subsequent divorce and the pain she felt:

The people that you allow into your hearts and that you know that you love and they have a special place for you and when they hurt or betray you or something, that level of hurt is, I mean it just devastated who I was, like I gave everything to that relationship.

#### 4.3.2 Finding the power to forgive through intrapersonal source or strategy

In describing the intrapersonal strategy that helped her to let go of anger towards God, Nina told of her experience of her brother dying and how running became a meditative outlet for her. “When my brother passed away, I was … really angry, you know, like ‘Why'd you take him?’” She said of running that it “was good because mentally it helps me, emotionally it helps me, but it's also healthy… and kind of became my outlet of going out and self‐reflecting.” Ben explained the importance of humility in his intrapersonal forgiveness process: “It's easier to forgive someone if you put their feelings into the equation, rather than your own. Sometimes you feel that you deserve forgiveness, rather than understanding what they feel and forgiving them, too.” Jean described the inner perspective that forgiveness is for yourself even if it does not improve an interpersonal relationship: “You can forgive, but that doesn't mean you have to … feel good about them, or let them in your life. That's why forgiving is a lot for yourself.”

#### 4.3.3 Finding the power to forgive through interpersonal source or strategy

Mary talked about how interpersonal boundaries were important in her forgiveness process. She described how after a person has offended her, she forgives but has the right to protect herself: “I can forgive you, but … now our relationship has changed.” She also described the forgiveness help she received from the influence of her family upbringing:

I was always taught you forgive. As hard as it is, like you forgive, you know. So, I think for me it's just like making sure that I'm okay spiritually, in my home life, and that will help me to be forgiving.

Reflecting on her relationship with her sister, Maria talked about the effect that important family relationships have on the forgiveness process for her:

I always forgive her, and she always forgives me, and it's just little things that may be said, but they hurt. … Because they're relationships that are super valuable, they're important, and you want to do right by it. So, I probably‐that would be my most struggle, and it's a constant struggle.

#### 4.3.4 Finding the power to forgive through transpersonal source or strategy

Barbara talked about her experience of the power of prayer on the night she was so angry at her husband that she literally was planning to kill him. Instead, she called her AA sponsor:

She told me to get down on my knees and pray to forgive him. I didn't want to, but I didn't want to end up in a correctional facility either. So I did what she asked. And the forgiveness came.

Vicky shared about her focus on enjoying the present moment as a strategy for transcending the effects of the offence and coming to forgiveness: “You don't pay attention to it. … I figure I don't have a whole lot of time left to spend it drudging up old feelings. … I just as soon enjoy today.” Margot described the transcendent power of music to help her cope with angry feelings: “Personally, music particularly allows me to get into a mindset that allows me to think more clearly (usually when I am angry with others or with myself Christian radio music helps).”

## 5 DISCUSSION

Our literature review revealed no lists of media for aiding the forgiveness process. This study provides evidence that media help people forgive and data which enabled us to create curated lists of media that may aid the forgiveness process. We consider this rich qualitative evidence of the power of media to aid the forgiveness process both profound and enlightening. As we interviewed the study participants, we felt honoured by how they shared deeply personal details of their forgiveness struggles with us. Some had made it through to the other side of inner turmoil and anger and others were still en route but all of them wanted to help in creating tools that could be helpful to others.

We discovered that this present study confirmed and added evidence to Recine et al. (2020). In that systematic review, the data from 11 nurse‐authored qualitative forgiveness studies led the first and second author in 2018 to create a handout of the findings, initially published on the first author's website, for use in the first and second authors' practices and as a tool for nurses in a Midwest hospice and palliative care unit, called “Guideposts to Forgiveness” (Recine et al., 2020, pp. 249–250). The 10 guideposts show how many people “changed their lives through forgiveness. These people were hurt by what often would seem unforgivable. They were justifiably angry but found the power to let go of that anger” (Recine et al., 2020, p. 249). The words of the guideposts “express the ideas and example of these people who found a way to live lives of peace and joy in spite of being hurt” (Recine et al., 2020, p. 249).

We examined the data from the present study, compared the coded transcripts with the findings reflected in the original 10 “Guideposts to Forgiveness” in Recine et al. (2020, pp. 249–250) and we were able to map the coded data from this present study to each category in the original “Guideposts to Forgiveness” findings (Recine et al., 2020, pp. 249–250) and contribute two new guideposts from the poignant descriptions of the participants in the present study. As a result of this present study, a revised *Guideposts to Forgiveness* (Recine & Recine, 2021) has been created, adding a new first and last guidepost (see Appendix 11). It is already being used as an educational tool by the first and second author at a Midwest university undergraduate class and in retreat settings for the lay public.

The two new findings revealed in the data from this present study that were not represented in the original 10 guideposts enhanced this resource. One new finding was experiencing the negative reality of the hurt. Discerning how the study participants were personally affected by, and aware of, wrongs became an important part of our analysis. The second new finding was how participants found their way to forgive themselves. Hence, guideposts for these have been added to the original 10. It is our hope that these two new guideposts will enhance the effectiveness of the original collection and be useful to nurses and other care providers in helping patients and clients find their way to the peace that forgiveness can bring.

Though the varied media our participants revealed were in English, we developed forgiveness‐related categories for the specific media mentioned, so that people in other cultures may find media in their own language which fit into these categories. These categories are based on data in the “Category Code” column in Appendices 1 and 3–10 (see below). It is our hope that in this way, our findings may bear fruit in a variety of cultures, internationally. We envision nurses and other care providers working with patients and clients to identify the type of media most suited to their individual needs on the forgiveness journey.

### 5.1 Internationally/cross‐culturally useful media categories

#### 5.1.1 Media for learning forgiveness through example

- Forgiveness exemplar‐general
- Forgiveness exemplar‐family forgiveness

#### 5.1.2 Media for learning forgiveness through educating oneself about the forgiveness process

- Self‐help
- Empathy‐enhancing about a [particular kind of person who hurt you]

#### 5.1.3 Media for becoming more forgiving towards yourself

- Self‐compassion
- Self‐forgiveness

#### 5.1.4 Media for becoming inspired and empowered to forgive others

- Inspirational
- Empowerment
- Inspirational‐religious

### 5.2 Strengths, limitations, and future directions

This study has strengths, but like many studies, it also has limitations. One strength is, that to the best of our knowledge, this study is the first to investigate what specific media and media types help people to forgive. Other strengths of this study are like the strengths of other qualitative studies, including the lengthy analysis period, weekly research team meetings with coding decisions arrived at by unanimous agreement, and 39 interviews that enabled the team to arrive at a point where no new themes were identified (data saturation) (see Sections 3.3 and 3.7 for more details). A limitation of the study is that all the media were in English. Even though many of the media are translated into other languages, practitioners in non‐English speaking cultures will need to take some effort to find translations or, using our internationally/cross‐culturally useful categories of media, to find suitable works from their own culture and in their own language. Other limitations are that most of the participants lived in the same region of the United States, were mostly white Christians, and there were twice as many female participants as males. Some participants had a great deal of media that they wanted to talk about that influenced them, others had a relative dearth of media that influenced them. We did not analyse our data with regards to social determinants that impact exposure to media, or through the lens of personal experience, exposure to the idea of forgiveness, culture, regions or age. This is a limitation of this study and a potentially fruitful focus for future research. Replication with a more internationally and culturally diverse population may enrich the present findings by revealing more types of media and forgiveness‐related media categories and additional forgiveness process themes.

Future research could include recruiting participants based on the severity and/or type of injustices experienced (perhaps on a Likert Scale). It is possible that this would have made a difference in the types of media that participants shared. Also, to address a possible limitation of the study, that participants may have shared general information about forgiveness rather than what helped them, future research could include a more explicit encouragement to take time to reflect on what media or types of media helped them to let go of negative feelings towards themselves or others and have a more kind intention towards themselves or others. A strength of the study is that though some participants seemed to be answering the questions spontaneously, many clearly came to the interview prepared. A future research design could also include the interviewers following up with all participants after the interview to receive additional media recommendations. Finally, our hope is that the updated *Guideposts to Forgiveness* (Recine & Recine, 2021) seen in Appendix 11, which capture the qualitative themes that inspire people to forgive, will be studied in future qualitative and quantitative studies.

## 6 CONCLUSION

Heideggerian Phenomenology is an excellent method to get at the depth of individual responses and to guide researchers in analysis. Regardless of age, gender or religious perspective, we found that individuals use media to help them forgive. This study provides new tools for health promotion through forgiveness facilitation. One is a collection of lists of diverse media (see Appendices 1–10) that may be useful to nurses and other healthcare providers in helping patients and clients in the forgiveness process. Another is an expanded version of *Guideposts to Forgiveness* (Recine & Recine, 2021) that can be used in nursing practice, university education and health promotion with the lay public (see Appendix 11). It is our hope that this expanded list of guideposts that represent the words and ideas of people in qualitative studies “who found a way to live lives of peace and joy in spite of being hurt” (Recine et al., 2020, p. 249) will be a tool that professionals around the globe could use with the same profound effect as we have found when we share them with hurting people.

We have begun the discovery of what kinds of media help people forgive. We challenge nurses and other professionals to continue the inquiry and, most importantly, to design action research that tests the usefulness of these tools to heal the bodies and minds of people who are struggling to let go of anger and find the peace that forgiveness can bring.

## AUTHOR CONTRIBUTIONS

AR designed the study. NW performed and analysed the literature review which informed the study's design. AA, LR, SH, MJ and SP collected the data. AR took the lead in the data analysis and AA, LR, SH, MJ and SP acted as co‐analysers. AR supervised the study. AR, LR, AA, SH, MJ and SP wrote the manuscript.

All authors have agreed on the final version and meet at least one of the following criteria [recommended by the ICMJE (http://www.icmje.org/recommendations/)]:

- substantial contributions to conception and design, acquisition of data or analysis and interpretation of data;
- drafting the article or revising it critically for important intellectual content.

## FUNDING INFORMATION

This research was graciously funded by grants from the University of Eau Claire‐Wisconsin's Office of Research and Sponsored Programs.

## CONFLICT OF INTEREST

No conflict of interest has been declared by the authors.

## Data Availability

The data sets generated during and/or analysed during the current study are not publicly available due to promised confidentiality of the participants.

## APPENDIX 1

### ADULT AGE BOOKS CITED AS INSPIRING FORGIVENESS

**Table:**

Title	Author	Publication year	Category code
*A Man Called Dave: A Story of Triumph and Forgiveness*	Dave Pelzer	2000	Forgiveness exemplar‐Family forgiveness
*Batman Three Jokers, Volume 3*	Marvel Comics	2020	Forgiveness‐Exemplar general
*Beauty for Ashes: Receiving Emotional Healing*	Joyce Meyer	1994	Inspirational‐Religious
*Daring Greatly: How the Courage to Be Vulnerable Transforms the Way We Live, Love, Parent, and Lead*	Brene̒ Brown	2012	Self‐Help
*Eat, Pray, Love*	Elizabeth Gilbert	2006	Self‐Compassion
*Educated: A Memoir* ^a^	Tara Westover	2018	Empathy‐Enhancing about a parent with severe mental illness
*From Forgiven to Forgiving: Learning to Forgive One Another God's Way*	Jay E. Adams	1994	Self‐Help
*Girl, Stop Apologizing: A Shame‐Free Plan for Embracing and Achieving Your Goals*	Rachel Hollis	2019	Empowerment
*Girl, Wash Your Face: Stop Believing the Lies About Who You Are so You Can Become Who You Were Meant to Be*	Rachel Hollis	2018	Empowerment
*I Thought It Was Just Me (but it isn't): Making the Journey from “What Will People Think?” to “I Am Enough”*	Brene̒ Brown	2007	Self‐Help
*Wish I Could Be There: Notes From a Phobic Life*	Allen Shawn	2007	Empathy‐Enhancing about a parent with agoraphobia
*Left to Tell: Discovering God Amidst the Rwandan Holocaust* ^b^	Immaculee Ilibagiza	2006	Forgiveness exemplar‐General
*Les Miserables*	Victor Hugo	1862	Forgiveness exemplar‐General
*Looking for Alaska*	John Green	2005	Self‐Forgiveness
*Milk and Honey* ^c^	Rupi Kaur	2014	Inspirational
*Morning and Evening*	Charles Spurgeon	1948	Inspirational‐Religious
*Next Level Thinking: 10 Powerful Thoughts for a Successful and Abundant Life*	Joel Osteen	2018	Self‐Help
*Nonviolent Communication: A Language of Life*	Marshall Rosenberg	1999	Self‐Help
*Pray Without Ceasing*	Wendell Berry	1992	Inspirational
*Rising Strong: How the Ability to Reset Transforms the Way We Live, Love, Parent, and Lead*	Brene̒ Brown	2015	Self‐Help
*The Amazing Spider‐Man Issue 38, 2013 Marvel Comics*	Marvel Comics	2013	Self‐forgiveness
*The Bait of Satan: Living Free From the Deadly Trap of Offense*	John Bevere	2012	Self‐Help
*The Battlefield of the Mind: Winning the Battle in Your Mind (Book and Study Guide)* ^d^	Joyce Meyer	1995	Self‐Help
*The Code of the Extraordinary Mind: 10 Unconventional Laws to Redefine Your Life and Succeed on Your Own Terms*	Vishen Lakhiani	2016	Self‐Help
*The Four Agreements*	Don Miguel Ruiz	2001	Self‐Help
*The Gifts of Imperfection: Let Go of Who You Think You're Supposed to Be and Embrace Who You Are*	Brene̒ Brown	2010	Self‐Help
*The Hiding Place* ^e^	Corrie ten Boom	1971	Forgiveness exemplar‐General
*The Lipstick Gospel: A Story About Finding God in Heartbreak, the Sistine Chapel, and the Perfect Cappuccino*	Stephanie May Wilson	2014	Empowerment
*The Path Made Clear: Discovering Your Life's Direction and Purpose*	Oprah Winfrey	2019	Inspirational
*The Pawnbroker*	Edward Lewis Wallant	1965	Forgiveness exemplar‐General
*The Perks of Being a Wallflower*	Stephen Chbosky	1999	Self‐Forgiveness
*The Prodigal God*	Timothy Keller	2008	Inspirational‐Religious
*The Subtle Art of Not Giving a F*ck: A Counterintuitive Approach to Living a Good Life*	Mark Manson	2016	Self‐Help
*When It Rains*	Maria Becker	2019	Empowerment

*Note*: *N* = 34.

^a^ Cited twice by participants.

^b^ Cited twice by participants.

^c^ Cited three times by participants.

^d^ Cited twice by participants.

^e^ Cited twice by participants.

## APPENDIX 2

### YOUTH BOOKS CITED AS INSPIRING FORGIVENESS

**Table:**

Title	Author	Publication year
Picture Books (Pre‐school – Grade 2)		
*Accident!*	Andrea Tsurumi	2017
*Caillou: Accidents Happen*	Anne Paradis	2014
*Desmond and the Very Mean Word: A Story of Forgiveness*	Desmond Tutu and Douglas Carlton Abrams	2012
*Forgiving a Friend*	Virginia Kroll	2005
*Horrible Bear!*	Anne Dyckman	2016
*Lilly's Purple Plastic Purse*	Kevin Henkes	2006
*Pete Makes a Mistake*	Emily McCully	2015
*Potato Pants*	Laurie Keller	2018
*Tell the Truth, B.B. Wolf*	Judy Sierra	2010
*The Berenstain Bears and the Forgiving Tree* ^a^	Jan Berenstain and Mike Berenstain	2011
*The Forgiveness Garden*	Lauren Thompson	2012
*The Grudge Keeper*	Mara Rockliff	2014
*The Voyage*	Veronica Salinas	2012
*We All Need Forgiveness*	Mercer Mayer	2014
*Where is My Balloon?*	Ariel Bernstein	2019
Middle Grade Books (Grade 3–6)		
*A Kind of Paradise*	Amy Tan	2019
*Circle of Secrets*	Kimberly Little	2011
*Lizzie Flying Solo*	Nanci Stevenson	2019
*Louisiana's Way Home*	Kate DiCamillo	2018
*Mascot*	Antony John	2018
*My Name is River*	Wendy Dunham	2015
*Restart*	Gordon Korman	2017
*Ten Rules for Living with My Sister*	Ann M. Martin	2012
*Train I Ride*	Paul Mosier	2017
Young Adult Books (Grade 7–12)		
*Hate List*	Jennifer Brown	2009
*Not if I Save You First*	Ally Carter	2018
*Red Sky At Morning*	Richard Bradford	2020
*Rumble*	Ellen Hopkins	2016
*See All Stars*	Kit Frick	2018
*Story of A Girl*	Sara Zarr	2017
*The Fetch*	Laura Whitcomb	2010
*The Lies About Truth*	Courtney Stevens	2017
*Touching Spirit Bear* ^b^	Ben Mikaelsen	2002
*What Light*	Jay Asher	2016
*Whirligig*	Paul Fleischman	2010

*Note*: *N* = 35. All titles except *Lily's Purple Plastic Purse*, *Red Sky at Morning*, *The Voyage*, and *We All Need Forgiveness* were provided by a participant who is a former librarian, in answer to the study's first research question: “What books, including religious or spiritual writings or other media could you suggest that could inspiresomeone to let go of anger toward themselves or others and have a more kind intention toward themselves or others?”

^a^ Cited twice by participants.

^b^ Cited twice by participants.

## APPENDIX 3

### MUSIC CITED AS INSPIRING FORGIVENESS

**Table:**

Song or album title	Artist	Release year	Category code
“7 times 70”	Chris August	2010	Forgiveness exemplar‐Family forgiveness
“Another in the Fire”	Hillsong United	2019	Inspirational‐Religious
“Be Alright”	Dean Lewis	2019	Inspirational
“Born This Way”	Lady Gaga	2011	Self‐Compassion
*Bring Your Nothing* (Album)	Shane and Shane	2013	Inspirational‐Religious
“Come and Fill Our Hearts”	Taizé Community	2006	Inspirational‐Religious
“Daddy Lessons”	Beyoncé	2016	Empathy‐Enhancing for an estranged father
“Forgiveness”	Matthew West	2012	Inspirational‐Religious
“KUN FAYAKUN”	A.R. Rahman, Javed Ali, Mohit Chauhan	2011	Self‐Forgiveness
“Last Hope”	Paramore	2013	Inspirational
*Lemonade* (Album)	Beyoncé	2016	Empowerment
“Lord Is It Mine”	Supertramp	1979	Inspirational‐Religious
“Mighty to Save”	Hillsong Worship	2006	Inspirational‐Religious
“Oceans”	Hillsong United	2013	Inspirational‐Religious
“Praying”	Kesha	2017	Empowerment
“Quiet”	MILCK	2018	Empowerment
“Taste and See”	James Moore Jr.	2001	Inspirational‐Religious
“This is Just so Beautiful”	Jenny and Tyler	2010	Inspirational
“Try”	Colbie Caillat	2014	Empowerment
“You are Mine”	David Haas	1996	Inspirational‐Religious
“You Loved My Heart to Death”	Shane and Shane	2013	Inspirational‐Religious
“Yours is No Disgrace”	Yes	1971	Self‐Compassion

*Note*: *N* = 22.

## APPENDIX 4

### POETRY, PRECEPTS AND PRAYERS CITED AS INSPIRING FORGIVENESS

**Table:**

Title	Source or author (if known)	Category code
*Footprints in the Sand*	Margaret Fishback Powers	Inspirational‐Religious
Lord, I am not worthy that you should enter under my roof, but only say the word and my soul shall be healed.	Excerpt, Invitation to Eucharist, Catholic Eucharistic Liturgy	Inspirational‐Religious
Prayer of St. Francis/Instrument of Your Peace	Attributed to St. Francis of Assisi	Inspirational‐Religious
Sorrowful Mysteries of the Rosary		Inspirational‐Religious
Steps 4–9 of Alcoholics Anonymous' Twelve Steps	Alcoholics Anonymous: The Story of How Many Thousands of Men and Women Have Recovered from Alcoholism, 2001, 4th ed.	Self‐Help
*Your Cross*	St. Francis de Sales	Inspirational‐Religious

*Note*: *N* = 6.

## APPENDIX 5

### SAYINGS CITED AS INSPIRING FORGIVENESS

**Table:**

Saying	Source or author (if known)	Category code
Even if I knew that tomorrow the world would go to pieces, I would still plant my apple tree today.	Attributed to Martin Luther	Inspirational
Forgiveness is the fragrance the violet sheds on the heel that has crushed it.	Attributed to Mark Twain	Inspirational
Forgiving does not erase the bitter past. A healed memory is not a deleted memory. Instead, forgiving what we cannot forget creates a new way to remember. We change the memory of our past into a hope for our future.	Attributed to Lewis B. Smedes	Self‐Help
Hurt people, hurt people. Forgiven people, forgive people.		Self‐Help
There are moments which mark your life. Moments when you realize nothing will ever be the same and time is divided into two parts: before this and after this. … Sometimes you can feel such a moment coming. That's the test, or so I tell myself. I tell myself at times like that, strong people keep moving forward anyway, no matter what they're going to find.	Attributed to Denzel Washington	Inspirational
To forgive is to set a prisoner free and discover that the prisoner was you.	Attributed to Lewis B. Smedes	Self‐Help
Unforgiveness (holding on to anger, bearing a grudge, resentment) is like drinking poison and expecting the other person to die.		Self‐Help
When we forgive evil we do not excuse it, we do not tolerate it, we do not smother it. We look the evil full in the face, call it what it is, let its horror shock and stun and enrage us, and only then do we (can we) forgive it.	Attributed to Lewis B. Smedes	Self‐Help
There's an important difference between forgiving a person and tolerating their bad behavior.	Attributed to Lewis B. Smedes	Self‐Help
What does not kill me makes me stronger.	Attributed to Friedrich Nietzsche	Inspirational
When you give up vengeance, make sure you are not giving up on justice. The line between the two is faint, unsteady and fine. …vengeance is our own pleasure of seeing someone who hurt us getting it back and then some. Justice, on the other hand, is secure when someone pays a fair penalty for wronging another even if the injured person takes no pleasure in the transaction. Vengeance is personal satisfaction. Justice is moral accounting. …Human forgiveness does not do away with human justice.	Attributed to Lewis B. Smedes	Self‐Help

*Note*: *N* = 11.

## APPENDIX 6

### MOVIES CITED AS INSPIRING FORGIVENESS

**Table:**

Title	Director; producer	Release year	Category code
*Amish Grace*	Dylan Scharping	2010	Forgiveness exemplar‐General
*Fireproof*	Alex Kendrick, Sherwood Pictures	2008	Forgiveness exemplar‐Family forgiveness
*Hacksaw Ridge*	Mel Gibson; David Permut, Terry Benedict, Paul Currie, Bruce Davey, et al.	2016	Forgiveness exemplar‐General
*I Can Only Imagine*	Andrew Erwin, Jon Erwin, Mission Pictures International	2018	Forgiveness exemplar‐Family forgiveness
*The Passion of the Christ*	Mel Gibson; Bruce Davey, Stephen McEveety	2004	Inspirational‐Religious
*Saving Private Ryan*	Steven Spielberg; Mark Gordon, Gary Levinsohn, Ian Bryce, Bonnie Curtis	1998	Self‐Forgiveness
*Shrek*	Andrew Adamson, Vicky Jenson; Aron Warner, John H. Williams, Jeffrey Katzenberg	2001	Forgiveness exemplar‐General
*Stan and Ollie*	Jon S. Barid; Faye Ward	2018	Forgiveness exemplar‐General
*The Elephant Man*	David Lynch; Jonathan Sanger	1980	Forgiveness exemplar‐General
*The Hiding Place*	James F. Collier; Frank R. Jacobson, William F. Brown	1975	Forgiveness exemplar‐General
*The Mission*	Roland Joffe; Fernando Ghia, Maximo Berrondo, David Puttnam	1986	Self‐Forgiveness
*The Shack*	Stuart Hazeldine; Gil Netter, Brad Cummings	2017	Inspirational‐Religious
*Toy Story 1*	John Lasseter; Bonnie Arnold Ralph Guggenheim	1995	Forgiveness exemplar‐General
*Unbroken*	Angelina Jolie, Jolie Pas, 3 Arts Entertainment, Legendary Entertainment	2014	Forgiveness exemplar‐General

*Note*: *N* = 14.

## APPENDIX 7

### PLAYS CITED AS INSPIRING FORGIVENESS

**Table:**

Title	Author	Release year	Category code
*August: Osage County*	Tracy Letts	2014	Empathy‐enhancing for a parent with severe mental illness
*Cyrano de Bergerac*	Edmond Rostand	1897	Forgiveness exemplar‐General
*Rabbit Hole*	David Lindsay‐Abaire	2006	Forgiveness exemplar‐Family forgiveness
*The Front Room* ^a^	Richard Kalinoski	Not released	Empathy‐enhancing for a parent with a hoarding diagnosis
*Wicked*	Stephen Schwartz	2003	Forgiveness exemplar‐General

*Note*: *N* = 5.

^a^Cited twice by participants.

## APPENDIX 8

### BIBLE PASSAGES CITED AS INSPIRING FORGIVENESS

**Table:**

Passage	Description	Category code
1 Corinthians 13	List of the characteristics of love. For example, love is patient, love is kind.	Inspirational‐Religious
1 John 2:1–2	Jesus is the atoning sacrifice for our sins.	Inspirational‐Religious
1 John 4:7–21	God's love inspires us to be loving.	Inspirational‐Religious
2 Corinthians 10:5	Mental discipline.	Inspirational‐Religious
2 Corinthians 12:9	God's grace and power in weaknesses.	Inspirational‐Religious
2 Samuel 12:1–14	God forgives King David.	Forgiveness exemplar
Ephesians 4:32^a^	Christ is a model of forgiveness.	Forgiveness exemplar
Galatians 6:2	Lean on someone.	Inspirational‐Religious
Genesis Chapters 37–45	Story of Joseph's forgiveness of his brothers.	Forgiveness exemplar
James 5:16	Power of prayer.	Inspirational‐Religious
John 16:33	Acceptance and courage.	Inspirational‐Religious
John 8:2–11	Jesus forgives a woman who committed adultery.	Forgiveness exemplar
Luke 12:48	Give forgiveness as you have received forgiveness.	Inspirational‐Religious
Luke 15:11–32^b^	A story of a father's forgiveness of his son.	Forgiveness exemplar‐Family forgiveness
Luke 23:33–34^c^	Jesus forgives those who crucify him.	Forgiveness exemplar
Luke 6:37	Promises about forgiveness.	Inspirational‐Religious
Matthew 18:21–22^d^	Forgive without limit.	Inspirational‐Religious
Matthew 18:23–35^e^	Parable about forgiving people from your heart because you are forgiven.	Inspirational‐Religious
Matthew 5:1–12	A description of what it is to be blessed.	Inspirational‐Religious
Psalms 118:13	God's grace and power in weaknesses.	Inspirational‐Religious
Romans 3:23	We're all sinners.	Inspirational‐Religious
Romans 8:26–30	God and the Holy Spirit working for us.	Inspirational‐religious
Romans 8:28	God can bring good out of evil.	Inspirational‐religious

*Note*: *N* = 34.

^a^ Cited three times by participants.

^b^ Cited twice by participants.

^c^ Cited twice by participants.

^d^ Cited four times by participants.

^e^ Cited twice by participants.

## APPENDIX 9

### QUR'AN AND OTHER ISLAMIC PASSAGES CITED AS INSPIRING FORGIVENESS

**Table:**

Passage	Description	Category code
Al Qur'an 15:85	A command to forgive graciously.	Inspirational‐Religious
Al Qur'an 2:117	The sovereignty of Allah.	Inspirational‐Religious
Al Qur'an 24:22	Imitate God's forgiveness.	Inspirational‐Religious
Al Qur'an 3:134	Pardoning others defines the believer.	Forgiveness exemplar‐General
Al Qur'an 42:40	Forgiveness is rewarded by God.	Inspirational‐Religious
Al Qur'an 42:43	Forgiving others is courageous.	Inspirational‐ Religious
Al Qur'an 45:14	Command from Allah to forgive unbelievers.	Inspirational‐Religious
Al Qur'an 7:199	Importance of showing forgiveness.	Inspirational‐Religious
Musnad Ahmad 16999 (Hadith)	Exhortation to pardon wrongs.	Inspirational‐Religious
Musnad Ahmad 7001 Hadith on Mercy	Allah forgives those who forgive others.	Inspirational‐Religious
Sahih Muslim 2588 (Hadith‐The book on Virtue)	Allah increases the honour of the one who forgives.	Inspirational‐Religious
Sunan At‐Tirmidhi 1424 (Hadith‐Book on Legal Punishments)	Mistake of being forgiving is better than punishing.	Inspirational‐Religious
Sunan At‐Tirmidhi 2016 (Hadith‐Chapters on Righteousness)	The Prophet is an example of pardoning others.	Forgiveness exemplar‐ General

*Note*: *N* = 13.

## APPENDIX 10

### VIDEOS AND PODCASTS CITED AS INSPIRING FORGIVENESS

**Table:**

Title	Speaker	Release year	Source	Category code
*The Power of Vulnerability*	Brene̒ Brown	2013	https://youtu.be/iCvmsMzlF7o	Empowerment
*Teacher of The Year*	Guy Doud	2017	https://youtu.be/mHUlaTOInOE (Part 1) https://youtu.be/AlygFbtXY7s (Part 2)	Inspirational
*What Hope Does God Offer in My Depression?*	John Piper	2018	https://www.desiringgod.org/interviews/what‐hope‐does‐god‐offer‐in‐my‐depression	Inspirational‐Religious

*Note*: *N* = 3.

## APPENDIX 11

### GUIDEPOSTS TO FORGIVENESS

Holistic Therapy LLC (Dr. Ann Recine) & Lou Recine Coaching LLC (Lou Recine), updated 2021 from the original 2018 version.

**Where the forgiveness guideposts come from**. The guideposts below come from interviews by nurses with many people who changed their lives through forgiveness. These people were hurt by what often would seem unforgivable. They were justifiably angry but found the power to let go of that anger. The words below express the ideas and example of these people who found a way to live lives of peace and joy in spite of being hurt.

1. **Understand your hurt**. Before you can forgive yourself or another person, you need to examine what happened to you—what led up to the event, who did what, and acknowledge that it was unjust, and hurt you. Then you need to recognize your feelings. Its ok to feel sad, lonely, irritable, anxious, or angry. It is important to understand how it is affecting your health, your body, energy level, concentration, or sleep.
2. **Begin with knowledge**. Know the definition of forgiveness. Forgiveness is letting go of your anger and resentment (even though justified) and choosing to have a positive intention towards the person who hurt you. It is not letting the person off the hook legally, nor is it forgetting, or pretending that it did not happen, nor deciding you are going to hang out and be friends. Forgiving does not mean giving up safety or personal boundaries.
3. **Press pause**. There are ways to learn how to gently stop the noisy circles of angry, painful thoughts in your mind. You can find a quiet place and time to calm yourself. Take time to figure out how you want to respond instead of reacting.
4. **Put your health first**. Know that the health of your body is connected to the health of your mind. Your health is more important than your hurt. Give yourself the gifts of health, peace of mind and happiness that comes from letting go of anger.
5. **Take baby steps**. Know that forgiveness and feeling better takes time. It starts with being *willing* to be willing to forgive. It begins with wanting to get your life back—with wanting to be at peace.
6. **Change channels**. We might not be in control of our thoughts initially, but we can learn to shift our focus. When you are angry or upset, you have the option to pick a more peaceful channel. You can think of what you are grateful for today. You can notice the pleasant and beautiful things in your life. You can focus on lifting someone else up today.
7. **Power‐up**. When your mind slips back into focusing on the hurt and hurtful person and you feel powerless over your angry feelings, reach out to a power greater than yourself. Notice what gives you strength and inspires hope that you can lead a happier, peaceful life in spite of having been hurt. Develop a practice of meditation, prayer, spiritual reading, religious services, or anything else that connects you to that source of power.
8. **Lean on somebody**. Do not be afraid to ask a strong, trustworthy person in your life for help. Choose to lean on people who care about you and are forgiving.
9. **See both sides**. Think about a time when you hurt someone in a way that was against your values. Deeply understand what was going through your mind and the pressures you were under. Seek to understand the kinds of pressures and struggles the person who hurt you was experiencing.
10. **Look for meaning**. There will come a day when you will be able to find the good that has come to you as a result of growing from the hurtful experience and even find the good in the other human being who hurt you. Let the virtues that you value help you make sense of the hurtful experience and achieve forgiveness as a personal victory.
11. **Realize life is short**. We do not know how many days we have left. Is there an attitude you would be willing to give up to have peace inside? It's empowering to take charge of a relationship by deciding to forgive. By choosing to forgive, you are showing people what you value and how you would like to be remembered.
12. **Forgive yourself**. You are human, vulnerable and you make mistakes. Self‐forgiveness is something you learn by understanding your own feelings and the feelings of others in a non‐judgmental way. Whether the cause of the trouble is your responsibility or someone else's, or a combination, you can approach yourself with compassion and a gentle determination to change for the better. That change may include a new pattern of behaviour, setting new boundaries and new ways to care for yourself and others.
